# Wood in the Wound: Detecting an Intra-Articular Wooden Foreign Body on Ultrasound

**DOI:** 10.1016/j.acepjo.2025.100200

**Published:** 2025-06-25

**Authors:** Christopher Smilios, Amanda Dalpiaz, Alexander Nello, Allison Cohen, Mathew Nelson

**Affiliations:** Department of Emergency Medicine, North Shore University Hospital, Manhasset, New York, USA

## 1 Patient Presentation

A 34-year-old man with no significant medical history presented to the emergency department with a 3-day history of right knee pain after falling on wood chips. Although he initially removed visible debris from the puncture site, he continued to experience pain with movement and ambulation. On presentation, he reported increasing swelling and erythema around the area.

## 2 Diagnosis: Wooden Foreign Body Penetrating The Joint

**Figure 1:**
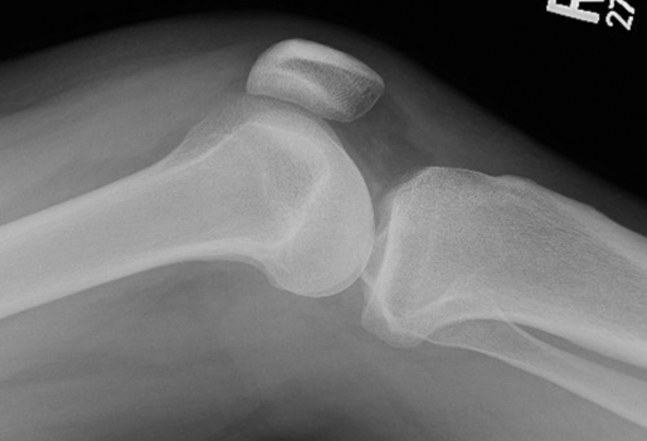
Lateral knee x-ray showing joint effusion with no visualized foreign body.

**Figure 2:**
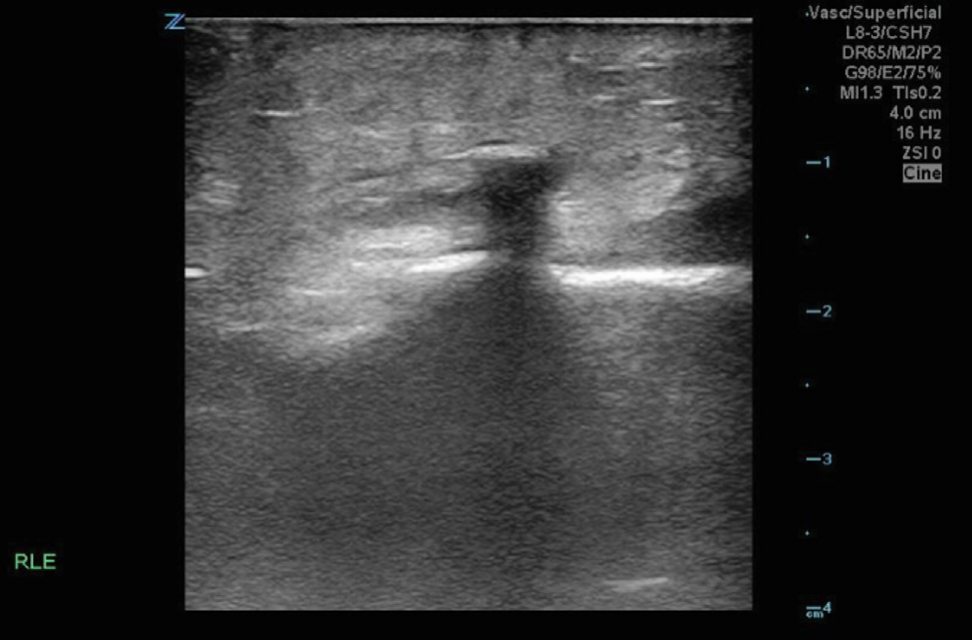
Linear hyperechoic foreign body visualized in the longitudinal plane with posterior acoustic shadowing consistent with a foreign body.

**Figure 3:**
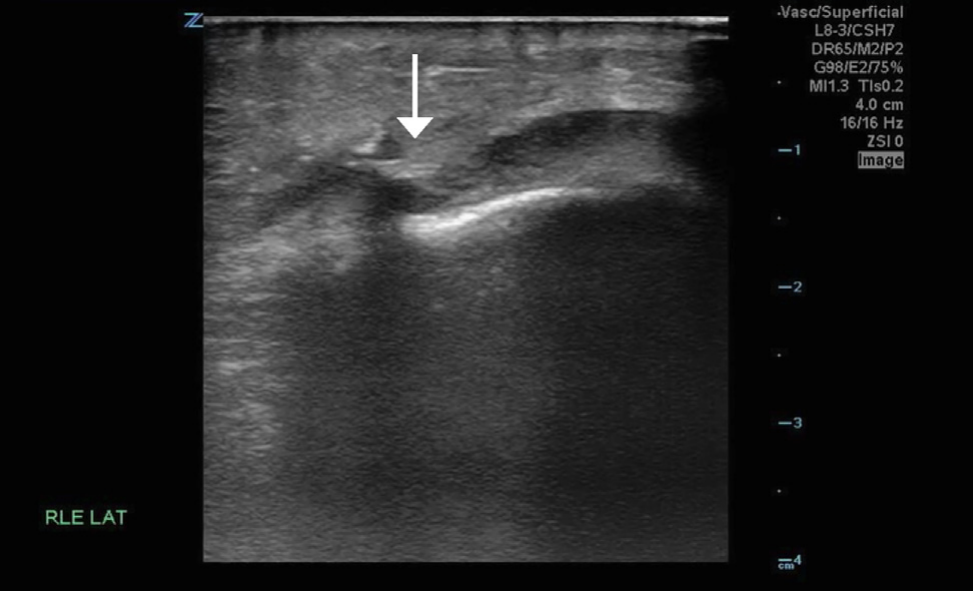
Radiopaque linear foreign body surrounded by hypoechoic synovial fluid, suggesting intra-articular intrusion. White arrow representing radiopaque linear foreign body surrounded by hypoechoic synovial fluid, suggesting intra-articular intrusion.

Initial knee radiographs revealed a joint effusion without evidence of a radiopaque foreign body (Fig 1). A computed tomography scan similarly failed to detect any radiopaque material or soft tissue gas. Synovial fluid analysis was inconclusive. Given persistent clinical concern, a point-of-care ultrasound (POCUS) was performed. POCUS revealed a linear echogenic structure with posterior shadowing, consistent with a wooden foreign body (Fig 2). The foreign body was visualized penetrating the joint capsule (Fig 3), and the patient was taken to the operating room for irrigation and debridement.

Wooden foreign bodies are notoriously difficult to detect on imaging. Only 7% to 15% are visible on plain radiographs, and computed tomography identifies just 63% to 70% overall.^1^, ^2^, ^3^ POCUS offers a radiation-free, accessible alternative with a reported sensitivity of 96.7% for detecting wooden foreign bodies.^4^^,^^5^ However, detection becomes more difficult over time, as wooden material becomes more isoechoic to surrounding tissue.^3^ In this case, POCUS was the only modality that visualized the foreign body and confirmed intra-articular extension, proving essential to timely diagnosis and management.

## Funding and Support

By *JACEP Open* policy, all authors are required to disclose any and all commercial, financial, and other relationships in any way related to the subject of this article as per ICMJE conflict of interest guidelines (see www.icmje.org). The authors have stated that no such relationships exist.

## Conflict of Interest

All authors have affirmed they have no conflicts of interest to declare.
